# The professional identity of Lithuanian midwifery students: An exploratory study

**DOI:** 10.18332/ejm/127515

**Published:** 2020-10-30

**Authors:** Alix Bukkfalvi-Cadotte

**Affiliations:** 1 Institut National de la Recherche Scientifique, Quebec, Canada

**Keywords:** profession, healthcare, midwifery, Lithuania, qualitative

## Abstract

**INTRODUCTION:**

Midwifery practice, which contributes to the improvement of the health and well-being of mothers and infants, varies greatly across the world. In former Soviet Union countries, midwifery was developed in a context marked by the Soviet regime and often remains underdeveloped. However, it is now gaining momentum in several countries including Lithuania where midwives have gained some autonomy in the past years. This study aims to explore the professional identity of student-midwives in Lithuania.

**METHODS:**

Semi-directed interviews were conducted with six student-midwives recruited in two educational institutions in Kaunas, Lithuania. The interviews addressed the respondents’ perception of midwifery in Lithuania as well as their personal experiences.

**RESULTS:**

This study suggests that the participants view midwifery as a medical profession, strongly anchored in the biomedical world. Indeed, they associate midwifery with medicine and nursing, they greatly value their medical degree, and they associate their profession with the hospital setting. The respondents further defined their profession by emphasizing their holistic woman-centred approach, which sets them apart from other healthcare professions. These elements of the participants’ professional identity can be viewed as professionalization strategies used to better establish their profession within the field of maternal healthcare.

**CONCLUSIONS:**

These findings suggest that the participant student-midwives view midwifery as a medical profession, rooted in the biomedical field, but also characterized by a unique care approach. This exploratory study thus contributes to a better understanding of midwifery in Lithuania.

## INTRODUCTION

Midwifery practice varies significantly across the world in terms of educational requirements and scope^1^. As evidence shows that midwifery contributes significantly to the improvement of the health and wellbeing of childbearing women and infants^2^, midwifery education and practice – as well as their variations across countries and time – constitute a crucial area of research.

In Lithuania, midwifery practice was developed in a political and social context marked by the Soviet regime, which favoured early and universal childbearing^3^ through pronatalist policies and the promotion of motherhood as a woman’s duty^4^. While healthcare coverage was widespread, the Soviet healthcare system was outdated and directed by centrally established guidelines, enforced throughout the Soviet Union regardless of local context^5^. Maternity care was characterized by an orientation towards technological management of birth, reflecting the trend towards standardization of care under the strict control of the state, with obstetricians holding authority and midwives assuming a supportive role^5,6^.

Midwifery practice remains underdeveloped in several former Soviet Union countries, where midwives face several challenges, including the lack of educational resources and insufficient legislative support^7,8^. However, midwifery is now gaining momentum throughout the region, especially with the increased availability of higher education programmes and the standardization of educational requirements^7,9^.

In Lithuania, maternal health has considerably improved in the past two decades; the proportion of pregnant women with no comorbidities has increased from 40% to 66%, and the maternal mortality ratio has dropped to an average of 6.26 for 100000 births for the 2005–2014 period^10^. The caesarean section rate reached 26.5% in 2012 before declining slightly in the following years^11^. Most deliveries in Lithuania take place in hospitals^11^. While home births were legalized in 2019^12^ and recent evidence shows that planned home births are not associated with higher maternal morbidity or infant mortality^13^, this practice remains rare in Lithuania.

Lithuanian midwives have gained a degree of autonomy in recent years, they can now provide independent antenatal care for low-risk pregnant women and have access to Bachelor’s degree-level midwifery education^9,12^. Midwifery practice in Lithuania as well as its larger social context are thus evolving, and students currently pursuing midwifery education in this country may provide a pertinent perspective on these ongoing transformations.

Conducted in partnership with the Cardiff University World Health Organization Collaborating Centre for Midwifery Development, this research project aims to explore the professional identity of Lithuanian student-midwives.

## METHODS

A qualitative methodology with an inductive approach was adopted for this study. Semi-directed interviews were conducted with six student-midwives recruited in two educational institutions in Kaunas, Lithuania. The criteria for selection were self-reported fluency in English, as the interviews were to be conducted in English, and having completed at least one year of their midwifery program. A convenience sampling method was used; the first three eligible students who expressed their interest after an in-person presentation at each educational institution were selected. The respondents were all women, aged 20–27 years, completing their second, third or fourth year of midwifery education at the time of the interview. The interviews were recorded and transcribed by the author, and all data was securely stored. Ethical approval was obtained from the Institut National de la Recherche Scientifique, Quebec, Canada.

The aim of the interviews was to identify common themes and concepts underlying the respondents’ professional identity through a thematic analysis of their answers to questions regarding: 1) motivations for becoming a midwife; 2) experience and perception of midwifery education and practice in Lithuania; and 3) understanding of interprofessional relations in the field of maternal healthcare.

## RESULTS

As detailed in Table 1, themes were grouped in two categories: themes related to a biomedical approach and those related to a holistic approach. These core elements of the participants’ professional identity can be analyzed as professionalization strategies used to legitimize and distinguish their profession in relation to other maternal care professionals.

**Table 1:** Themes related to participants’ professional identity

*Approach*	*Themes*	*Summary of findings*
**Biomedical approach**	Midwifery as a medical profession	Respondents appear to view midwifery as a medical profession.
	Medical education	The medical education of midwives is highly valued by respondents.
	Hospital setting	Respondents seem to associate midwifery and childbirth with the hospital setting.
	**Holistic approach**	Holistic perception of childbirth	Respondents see childbirth as a holistic, multidimensional experience.
Care work		Respondents emphasize the care work of midwives.

### Biomedical approach

The findings reveal that the participants view midwifery as a profession anchored in the biomedical world.

#### Midwifery as a medical profession

Several aspects of the participants’ statements seem to indicate that they define midwifery as a medical profession. For example, participants often categorized midwifery with the medical professions of medicine and nursing; one explained that a midwife is ‘the part between doctor and nurse, it's like in the middle.’ (A2). Four out of six respondents stated that they originally wanted to become doctors and perceived midwifery as a comparable profession. For the participants, midwifery thus appears to be firmly positioned within the biomedical professional sphere and defined as a medical profession.

#### Medical education

The participants also appeared to legitimize their practice by emphasizing the value of their midwifery degree, obtained through the nursing or medical faculty. This distinguishing feature was most often mentioned in relation to other maternity care professionals who do not hold a related degree such as doulas – attendants who provide non-medical assistance to childbearing women. Half of the respondents specifically referred to the differences between midwives’ and doulas’ educational backgrounds; for instance, a student-midwife said that ‘midwives and doulas don't get along because, well, we have a medical degree and they don't.’ (B1).

#### Hospital setting

The participants seemed to associate midwifery and childbirth with the hospital setting, rather than private homes. Indeed, five out of six respondents expressed concerns or insecurities concerning home births, including one who mentioned that home births are not common ‘because it's hard to find a midwife who would deliver a child at home […] because all the equipment is in the hospitals and you don't have it at home, and the midwives are just afraid of losing the baby and all the responsibility.’ (B2). These results suggest a biomedical view of childbirth focused on potential medical interventions and hospital-based risk management.

The interviews reveal that the student-midwives who participated in this research view midwifery as a medical profession, legitimized by a biomedical degree, and strongly associated with the hospital setting.

### Holistic approach

The participants also described a holistic woman-centred perspective; they distinguished their practice from that of other medical professionals, such as doctors and nurses, by emphasizing their unique perspective on pregnancy and childbirth and their care work.

#### Holistic perception of childbirth

Two respondents contrasted the midwives’ and doctors’ view of childbirth, one explaining that ‘midwives usually see it more like a happy experience […] a normal thing the human body does. And doctors usually see the mechanic action.’ (A3). Midwifery thus seems to be characterized by a unique perspective on childbirth, which is seen as a woman’s physiological and emotional experience in its globality.

#### Care work

The participants’ holistic view of childbirth was translated into their practice as midwives-in-training through providing emotional support. Two mentioned the privileged relationship between the midwife and the mother, fostered by extended contact, communication, and understanding. Thus, care work bears great importance in the professional identity of the respondents; they value their distinctive role in supporting mothers.

## DISCUSSION

This exploratory study suggests that the participants’ professional identity is particularly rooted in the biomedical world. However, this strong biomedical facet is complemented by a unique woman-centred approach to care, which distinguishes midwifery from other medical professions.

The remarks made by the student-midwives regarding the characteristics of their profession and their relationship to other professionals in their field can be viewed as strategies of professionalization^14,15^, used to legitimize a profession and secure its scope of practice. For instance, the focus on the medical dimension of the midwifery education and practice can be viewed as both an inclusionary and an exclusionary strategy^15^; it allows midwives to be included in the medical management of childbirth along with doctors and nurses, while excluding non-medical professionals, such as doulas, from their practice. The ideas expressed by the participants can thus reflect larger tendencies in interprofessional dynamics in the field of maternal healthcare, revealing how different professional groups define themselves and relate to one another.

### Strengths and limitations

This remains an exploratory study. The limited data obtained from only six interviews must be regarded critically and should be confirmed and enriched by further study. However, this research provides avenues for reflection regarding student-midwives’ professional identities in Lithuania and contributes to a better understanding of local variations in the field of maternal care. This research will thus allow researchers and practitioners to develop and improve their context-sensitive and reflexive approach in studying and supporting midwifery throughout Europe.

## CONCLUSIONS

This exploratory study suggests that the participating student-midwives’ professional identity is strongly associated with the biomedical field, but also with a distinct woman-centred approach to care. These results contribute to a better understanding of midwifery practice and interprofessional dynamics in the field of maternal care in Lithuania. A quantitative study involving a larger sample of student-midwives is needed to explore the representativity of these findings. Further research examining these themes through qualitative studies involving practicing midwives, other healthcare professionals, and childbearing women from varying backgrounds, could enrich these findings.
